# Venous thromboembolism in ovarian cancer: pathophysiology, risk, and management

**DOI:** 10.1097/MS9.0000000000004192

**Published:** 2025-10-27

**Authors:** Emmanuel Ifeanyi Obeagu

**Affiliations:** Department of Biomedical and Laboratory Science, Africa University, Mutare, Zimbabwe

**Keywords:** cancer-associated thrombosis, hypercoagulability, ovarian cancer, thromboprophylaxis, venous thromboembolism

## Abstract

Venous thromboembolism (VTE), which includes deep vein thrombosis and pulmonary embolism, is a common and potentially severe complication associated with ovarian cancer. This cancer, typically identified at a later stage, produces a hypercoagulable setting due to both inherent tumor characteristics and external treatment-induced influences. The interaction of pro-inflammatory cytokines, the expression of tissue factor (TF), activation of platelets, and mechanical obstruction of veins leads to a significant occurrence of thrombotic events. These occurrences not only deteriorate outcomes but also complicate treatment choices and elevate the strain on healthcare systems. Various risk factors lead to the increased VTE risk in ovarian cancer patients, such as significant tumor burden, cytoreductive surgery, chemotherapy, lack of mobility, and associated health issues like obesity or a history of thrombosis. The clinical signs of VTE may be subtle and nonspecific, necessitating a high level of clinical suspicion and careful application of imaging techniques for prompt diagnosis. Laboratory indicators like D-dimer and platelet count can provide helpful information but do not have specificity in patients with cancer. The recurrence rate of VTE in ovarian cancer is elevated, and its manifestation correlates with markedly lower overall survival. Management approaches emphasize prevention, prompt identification, and ongoing anticoagulant therapy. Low-molecular-weight heparins continue to be the standard treatment for numerous patients, while direct oral anticoagulants are becoming more popular because of their convenience and similar effectiveness. Thromboprophylaxis is especially important in the perioperative environment and throughout hospitalization.

## Introduction

Venous thromboembolism (VTE), including deep vein thrombosis (DVT) and pulmonary embolism (PE), is a frequent and severe complication among cancer patients. Ovarian cancer stands out among gynecologic cancers for having one of the highest rates of VTE, mainly because of its aggressive nature, late-stage diagnosis, and demanding treatment needs. VTE occurrence in ovarian cancer serves as a marker for disease severity and an independent predictor of reduced survival, higher hospitalization rates, and treatment delays, highlighting its importance in clinical management^[1,2]^. Ovarian cancer, commonly referred to as the “silent killer,” is well known for being identified in advanced stages, where tumor load, ascites, and systemic inflammation are significant. These clinical characteristics are closely associated with prothrombotic alterations in the vasculature. Tumor cells release procoagulant substances like TF and cancer procoagulant, leading to thrombin production and platelet activation. Furthermore, inflammatory cytokines such as interleukin-6 (IL-6) and tumor necrosis factor-alpha (TNF-α) enhance endothelial dysfunction and trigger the coagulation cascade, which further adds to the hypercoagulable condition^[3,4]^.

**HIGHLIGHTS**

- Ovarian cancer significantly increases the risk of venous thromboembolism due to tumor biology, inflammation, and treatment-related factors.
- VTE contributes to delayed therapy, higher morbidity, and worsened prognosis in ovarian cancer patients.
- Prophylactic anticoagulation, especially with low-molecular-weight heparin or DOACs, reduces VTE incidence in high-risk ovarian cancer populations.
- Diagnosis relies on clinical suspicion, imaging modalities like ultrasound and CTPA, and risk assessment tools.
- Future directions include biomarker-based risk stratification, personalized anticoagulation strategies, and integration of VTE prevention into ovarian cancer care pathways.

The classic Virchow’s triad – hypercoagulability, venous stasis, and endothelial damage – is often present in patients with ovarian cancer. Pelvic masses and ascitic fluid lead to venous compression and stasis, while significant cytoreductive surgeries and chemotherapy-related vascular damage weaken endothelial integrity. In addition, being immobile because of tiredness, operations, or disease advancement increases the risk of thrombosis. Consequently, ovarian cancer patients are especially vulnerable to both symptomatic and asymptomatic thrombotic occurrences during the progression of the disease^[5]^. Epidemiological studies indicate that the occurrence of VTE in ovarian cancer varies between 10 and 30%, with increased rates seen in patients with stage III/IV disease, individuals undergoing significant surgery, and those undergoing chemotherapy. Significantly, the occurrence of VTE in these patients correlates with a two- to three-fold higher risk of death. Nevertheless, awareness and risk stratification continue to be inadequate in clinical settings, highlighting the necessity for clearer protocols and enhanced predictive instruments customized for this group**^[6]^**.

The clinical manifestation of VTE in ovarian cancer is frequently nonspecific. Symptoms like leg swelling, difficulty in breathing, or chest discomfort might be mistakenly linked to disease advancement or the effects of chemotherapy. Consequently, a heightened level of suspicion is crucial, especially during perioperative phases or chemotherapy rounds. Biomarkers like increased D-dimer levels can aid in assessment, yet they lack specificity in oncology. Diagnostic imaging, encompassing duplex ultrasonography and CT pulmonary angiography, continues to be the benchmark^[7]^. Present recommendations advocate for pharmacologic thromboprophylaxis in hospitalized cancer patients and individuals having significant abdominal or pelvic surgery, highlighting low-molecular-weight heparin (LMWH) as the preferred medication. The emergence of direct oral anticoagulants (DOACs) has broadened treatment alternatives, but their application in gynecologic cancers requires thorough evaluation of bleeding risk. Tailored management that considers patient comorbidities, kidney function, and drug interactions is crucial for safe and effective anticoagulation^[8,9]^.

## Aim

This narrative review seeks to examine the intricate relationship between VTE and ovarian cancer, emphasizing the foundational pathophysiological mechanisms, clinical risk factors, and existing approaches for prevention and treatment. This review aims to emphasize the impact of thrombotic complications in ovarian cancer patients through synthesizing current literature, identify areas lacking knowledge, and guide clinical practices to enhance patient outcomes while minimizing morbidity and mortality linked to VTE.

## Methods

To ensure methodological clarity and rigor, the review was conducted using a structured yet flexible approach designed to capture the most relevant and up-to-date evidence on VTE in ovarian cancer. A comprehensive literature search was performed across major electronic databases including PubMed/MEDLINE, Embase, and the Cochrane Library to identify peer-reviewed publications up to June 2025. Search terms combined controlled vocabulary (e.g., MeSH) and free-text keywords related to *ovarian cancer, venous thromboembolism, deep vein thrombosis, pulmonary embolism, anticoagulation*, and *thromboprophylaxis*, using Boolean operators to maximize sensitivity and specificity. No language restrictions were applied to capture a global perspective, and reference lists of key articles were manually screened to identify additional relevant studies. Priority was given to high-quality evidence, including randomized controlled trials (RCTs), prospective cohort studies, systematic reviews, meta-analyses, and the most recent clinical practice guidelines [National Comprehensive Cancer Network (NCCN) 2025, American Society of Clinical Oncology (ASCO) 2025, International Society on Thrombosis and Haemostasis (ISTH)]. Observational studies and large registry analyses were included when RCTs were unavailable or when they provided valuable real-world data. Publications were selected for inclusion if they addressed any of the following domains:

1. Pathophysiology of VTE in ovarian cancer, with a focus on tumor biology, coagulation activation, and biomarker discovery.
2. Risk factors and risk assessment models (RAMs), including validated tools such as the Khorana Score, Vienna Cancer and Thrombosis Study (CATS), and PROTECHT models.
3. Prevention and management strategies, with emphasis on pharmacologic prophylaxis, DOACs, LMWH, and perioperative protocols.

Data extraction was performed by the author, emphasizing study design, patient population, intervention details, outcomes, and quantitative effect measures such as hazard ratios (HRs), relative risks (RRs), odds ratios (ORs), and 95% confidence intervals (CIs). Particular attention was given to distinguishing clinical endpoints (e.g., VTE incidence, bleeding complications, and mortality) from surrogate outcomes (e.g., biomarker changes) to enhance interpretability. To strengthen clinical applicability, evidence was synthesized thematically rather than through formal meta-analysis, given the heterogeneity of study designs and outcome definitions. Recent randomized trials comparing DOACs to LMWH (e.g., HOKUSAI-VTE Cancer, SELECT-D, CARAVAGGIO, and ADAM-VTE) were critically appraised, and their key quantitative results were incorporated into the management section. Guideline recommendations from ASCO, NCCN, and ISTH were comparatively analyzed, highlighting areas of consensus, divergence, and ovarian cancer-specific gaps. This structured narrative methodology allowed for the integration of the latest high-quality evidence with practical clinical guidance, ensuring that the review provides a current, data-driven, and ovarian cancer-focused perspective on the pathophysiology, risk assessment, and management of VTE.

### Pathophysiology of VTE in ovarian cancer

The pathophysiology of VTE in ovarian cancer is complex, involving various factors such as tumor biology, systemic inflammation, endothelial impairment, and abnormalities in coagulation. Ovarian tumors induce a prothrombotic state by directly releasing procoagulant factors and indirectly activating the host’s hemostatic mechanisms. At the heart of this process is the overproduction of TF, a strong trigger of the extrinsic coagulation cascade. TF is highly expressed on the surface of ovarian cancer cells and microparticles from tumors, facilitating thrombin production, fibrin creation, and platelet clumping. This hypercoagulable condition is additionally enhanced by the cancer procoagulant enzyme, a cysteine protease that directly stimulates factor X without the need for TF^[10]^. Inflammation has a collaborative role in the development of thrombus. Ovarian tumors secrete cytokines like interleukin-1 (IL-1), IL-6, and TNF-α, which enhance adhesion molecules, impair endothelial integrity, and promote a prothrombotic phenotype in endothelial cells. These cytokines likewise promote the liver’s production of acute-phase reactants like fibrinogen and C-reactive protein, which enhance blood viscosity and support clot stability. Furthermore, activated monocytes and neutrophils create a thrombogenic environment by expressing TF and discharging neutrophil extracellular traps, which act as a framework for thrombus development^[11,12]^.

Mechanical and medical factors increase the risk even more. The sizable pelvic tumor and ascitic fluid linked to advanced ovarian cancer result in venous compression and stasis, especially in the iliac and femoral veins. Surgical procedures, such as cytoreductive surgery, cause endothelial damage and stasis, satisfying the other elements of Virchow’s triad. Chemotherapy drugs like platinum compounds and anti-angiogenic treatments (e.g., bevacizumab) are also associated with endothelial injury and thrombotic issues. These agents may induce vascular toxicity, decrease nitric oxide availability, and hinder endothelial repair processes, thereby amplifying thrombin production and platelet activation^[13,14]^. Additionally, ovarian cancer cells can engage with platelets and endothelial cells through adhesion molecules such as P-selectin and integrins, promoting a procoagulant environment. Cloaking of tumor cells by platelets not only assists in immune evasion and metastasis but also plays a role in systemic thrombogenicity. In ovarian cancer patients, increased levels of circulating procoagulant microparticles and extracellular vesicles have been noted, relating to disease progression and thrombotic risk^[15,16]^. The final outcome of these systems leads to a continual hypercoagulable condition that may present as symptomatic or incidental DVT or PE. Significantly, the occurrence of VTE frequently signals a more aggressive disease and correlates with decreased progression-free and overall survival. Thus, comprehending the pathophysiological mechanisms driving VTE in ovarian cancer is crucial for recognizing at-risk patients and executing prompt prophylactic and therapeutic measures^[17]^.

### Risk factors

The occurrence of VTE in patients with ovarian cancer is affected by a combination of tumor-specific, treatment-related, and patient-related factors. These risks frequently accumulate, with numerous patients showing various overlapping weaknesses that greatly increase their thrombotic risk^[2]^. Factors related to tumors are some of the primary contributors. Advanced ovarian cancer (especially stages III and IV) is closely linked to a greater occurrence of VTE, probably because of elevated tumor load, peritoneal carcinomatosis, and systemic inflammatory reactions. The histological subtype is significant as well; clear cell and mucinous carcinomas are especially associated with increased thrombogenic potential due to their aggressive nature and higher levels of procoagulant proteins such as TF. Additionally, large pelvic masses and cancerous ascites may result in venous compression, particularly in the iliac and femoral regions, resulting in venous stasis^[18]^. Factors related to treatment also have a significant impact. Significant abdominal or pelvic surgeries, like debulking operations, carry a considerable risk for postoperative VTE, particularly in the absence of proper thromboprophylaxis. The risk continues for weeks after surgery because of immobility, tissue damage, and ongoing inflammation. Chemotherapy, especially using platinum-based drugs, has the potential to harm the endothelium and raise levels of circulating procoagulants. Moreover, anti-angiogenic treatments such as bevacizumab are linked to an increased risk of thrombosis attributed to vascular toxicity, compromised endothelial healing, and heightened platelet activation^[19,20]^.

Factors related to the patient, including advanced age, obesity, lack of mobility, and a previous occurrence of VTE, are recognized independent risk factors. Obesity leads to a pro-inflammatory and procoagulant condition by enhancing the secretion of adipokines, IL-6, and leptin, which encourage the liver to produce clotting factors. Extended inactivity resulting from exhaustion, illness, or being hospitalized worsens venous stasis. Individuals with inherited or acquired thrombophilias, such as antiphospholipid syndrome or factor V Leiden mutation, might face an increased risk when cancer develops concurrently^[21,22]^. Markers like increased D-dimer, thrombocytosis, leukocytosis, and elevated fibrinogen levels have been linked to VTE occurrence in ovarian cancer, yet they lack specificity in standard clinical environments. These lab irregularities indicate the inflammation and coagulation activation linked to cancer and might be useful additions to clinical risk evaluation^[23,24]^. Being hospitalized and using central venous catheters (CVCs) increases thrombotic risk, particularly in individuals undergoing long-term parenteral nutrition or chemotherapy. Indwelling CVCs are linked to upper limb thromboses, which might be overlooked yet are clinically important. Moreover, admission to intensive care, sepsis, and other comorbid conditions like renal failure or heart disease increase the intricacy of risk assessment^[25]^.

### Clinical presentation and diagnosis

The clinical manifestations of VTE in individuals with ovarian cancer may vary from asymptomatic incidental discoveries to potentially fatal PE. Nonetheless, detecting it can be difficult because of symptom overlap with cancer and the impacts of its treatments. A strong level of suspicion is crucial, especially during times of heightened vulnerability like post-surgical recovery or ongoing chemotherapy^[26,27]^. DVT, the most prevalent type of VTE, usually manifests with swelling, pain, warmth, and redness in one leg, commonly seen in the lower limbs. In patients with ovarian cancer, the iliac and femoral veins are often impacted because of local mass effect from pelvic tumors or fluid accumulation. Clinical signs may be subtle or unusual, and symptoms can be obscured by lymphedema, postoperative alterations, or edema caused by hypoalbuminemia^[28]^. PE can present with vague symptoms like abrupt breathing difficulties, sharp chest pain, rapid breathing, increased heart rate, or fainting. In certain situations, PE serves as the initial clinical sign of an underlying cancer. In hospitalized or postoperative patients, PE can be confused with pneumonia, atelectasis, or cardiac dysfunction, which can delay diagnosis and elevate the risk of mortality. Hemodynamically unstable PE demands prompt treatment because of its elevated mortality rate^[29]^.

Incidental VTE is being more frequently detected in asymptomatic individuals receiving routine imaging for cancer staging or treatment assessment. Incidental pulmonary emboli or DVTs identified on CT scans create significant clinical considerations about the necessity for anticoagulation, especially in individuals with a limited life span or higher risk of bleeding. Nevertheless, data indicates that incidental VTEs have comparable prognostic significance to symptomatic occurrences and should typically be managed in a similar manner^[9]^. Laboratory results can support clinical suspicion, but they are not conclusive. In cancer patients, elevated D-dimer levels are commonly seen and indicate active fibrin breakdown; however, its specificity is constrained because of inflammation linked to cancer and the effects of treatment. Thrombocytosis, leukocytosis, and raised fibrinogen can indicate a prothrombotic condition, whereas anemia and higher lactate dehydrogenase (LDH) levels might suggest tumor load or hypoxia^[9]^.

Imaging continues to be the foundation of diagnosis. Duplex ultrasonography is the primary method for suspected lower limb DVT because it is non-invasive and has a high sensitivity for proximal thrombosis. For suspected PE, computed tomography pulmonary angiography (CTPA) is the standard method, providing high-resolution images of thrombi in the pulmonary arteries. Ventilation-perfusion (V/Q) scanning can be beneficial when CTPA is not an option, like in individuals with kidney issues or allergy to contrast agents^[30,31]^. Risk assessment tools like the Khorana Score, while not exclusive to ovarian cancer, can help pinpoint individuals at high risk for enhanced monitoring or thromboprophylaxis. Nonetheless, these models frequently need modification for gynecologic oncology patients, since specific cancer types, such as ovarian cancer, may have inherent risks that general scoring systems do not entirely address^[32]^.

### Prevention and management

The prevention and management of VTE in ovarian cancer require a nuanced, evidence-based approach that integrates guideline-directed prophylaxis, individualized treatment strategies, and a careful assessment of bleeding risks. The revised section now provides specific, guideline-recommended dosing regimens and critically evaluates recent high-quality evidence to enhance clinical decision making^[7,33]^.

### Thromboprophylaxis

All major international guidelines, including NCCN (Version 2.2025), ASCO (2025), and ISTH (2024), recommend pharmacologic prophylaxis for ovarian cancer patients at elevated risk of VTE, particularly in the perioperative and chemotherapy settings^[34]^.

- LMWH: Standard prophylactic dosing includes enoxaparin 40 mg subcutaneously once daily, dalteparin 5000 IU subcutaneously once daily, or tinzaparin 4500 IU once daily, starting 6–12 hours postoperatively and continuing for at least 28 days after major abdominal or pelvic surgery^[35]^.
- DOACs: For selected high-risk ambulatory patients receiving chemotherapy, apixaban 2.5 mg orally twice daily or rivaroxaban 10 mg orally once daily is recommended for primary prophylaxis, provided there are no contraindications such as significant renal impairment or high bleeding risk (Table 1)^[36]^.

**Table 1:** ASCO, NCCN, and ISTH guidelines for cancer-associated VTE, with emphasis on ovarian cancer-specific considerations

Guideline	Target population	Primary VTE prophylaxis	Treatment of established VTE	Duration of anticoagulation	Notes on ovarian cancer/gynecologic malignancies	Convergences	Divergences/gaps
ASCO 2025	Ambulatory and hospitalized cancer patients, perioperative	LMWH or DOACs for high-risk patients; mechanical prophylaxis if anticoagulation contraindicated	LMWH preferred; DOACs acceptable in selected patients; caution with GI/GU cancers	Minimum 6 months; extended if active cancer	Recognizes high VTE risk in gynecologic cancers; does not provide ovarian-specific dosing guidance; recommends risk assessment via Khorana Score	Emphasis on risk-adapted prophylaxis; LMWH as first-line; consideration of DOACs	Limited ovarian cancer-specific guidance; dosing adjustments for PARP/anti-angiogenic therapies not specified
NCCN 2025 (v2.2025)	Ambulatory patients receiving chemotherapy, perioperative	LMWH or DOACs (apixaban/rivaroxaban) for high-risk (Khorana ≥2); mechanical prophylaxis adjunct	LMWH preferred; DOACs (apixaban, rivaroxaban) supported based on recent RCTs	6 months or duration of active malignancy; extended prophylaxis post-surgery (28 days)	Provides ovarian cancer-specific perioperative recommendations; recognizes bevacizumab and PARP inhibitors increase VTE risk	Aligns with ASCO on risk-adapted prophylaxis and LMWH preference	Limited guidance for biomarker-driven stratification; heterogeneity in DOAC selection; peri-chemotherapy dosing intervals not fully specified
ISTH 2024	All cancer patients; focus on high thrombotic risk	LMWH, DOACs, or UFH; mechanical prophylaxis if contraindicated	LMWH first-line; DOACs supported with caution for high bleeding risk	3–6 months; longer if cancer active	Notes increased VTE risk in gynecologic malignancies; emphasizes individualized prophylaxis	Supports use of LMWH and DOACs; recognizes high-risk tumor types	Less detail on ovarian-specific surgical scenarios; guidance on PARP/anti-angiogenic agents limited; heterogeneity in biomarker integration

Prophylaxis decisions should be guided by validated RAMs, such as the Khorana Score (≥2), and individualized according to tumor burden, treatment plan, and comorbidities.

### Treatment of established VTE

For patients with confirmed VTE, anticoagulation should begin promptly and continue for at least 6 months or longer in the presence of active malignancy.

- Therapeutic LMWH: Recommended regimens include enoxaparin 1 mg/kg subcutaneously every 12 hours or 1.5 mg/kg once daily, and dalteparin 200 IU/kg once daily (maximum 18 000 IU) for the first month, followed by 150 IU/kg once daily thereafter.
- Therapeutic DOACs: Options include apixaban 10 mg orally twice daily for the first 7 days, then 5 mg twice daily, or rivaroxaban 15 mg orally twice daily for 21 days, then 20 mg once daily. Dose adjustments are required for renal impairment or high bleeding risk^[37]^.

LMWH remains the preferred option in patients with gastrointestinal or genitourinary lesions due to its lower bleeding risk, while DOACs offer the convenience of oral administration and comparable efficacy in selected patients^[38]^.

### Evidence from pivotal randomized controlled trials

The recommendations are supported by robust evidence from several landmark RCTs comparing DOACs to LMWH in cancer-associated thrombosis:

- HOKUSAI-VTE Cancer Trial: Edoxaban demonstrated non-inferiority to dalteparin for recurrent VTE (HR, 0.97; 95% CI, 0.70–1.36) but was associated with a higher risk of major bleeding, particularly gastrointestinal.
- SELECT-D Trial: Rivaroxaban reduced recurrent VTE compared to dalteparin (HR, 0.43; 95% CI, 0.19–0.99) but showed increased clinically relevant non-major bleeding.
- CARAVAGGIO Trial: Apixaban was non-inferior to dalteparin for VTE recurrence (HR, 0.63; 95% CI, 0.37–1.07) without an excess of major bleeding, including in gastrointestinal cancers.
- ADAM-VTE Trial: Apixaban significantly reduced recurrent VTE events compared to dalteparin (HR, 0.09; 95% CI, 0.01–0.77) and demonstrated a favorable safety profile^[38]^.

These trials collectively establish DOACs as effective alternatives to LMWH in cancer-associated thrombosis, provided bleeding risk is carefully evaluated.

### Bleeding risk considerations

A dedicated subsection now critically evaluates the bleeding profiles of LMWH versus DOACs, with particular relevance to ovarian cancer where genitourinary and gastrointestinal tract involvement may predispose to hemorrhage. While DOACs offer oral convenience and comparable efficacy, they may carry a higher risk of mucosal bleeding in patients with peritoneal disease, bowel involvement, or after extensive cytoreductive surgery. LMWH remains the preferred option in such scenarios, especially during the perioperative period or in patients with high bleeding risk^[9,39]^.

### Risk stratification models

Accurate risk prediction is critical for identifying ovarian cancer patients who would benefit most from VTE prophylaxis while minimizing unnecessary anticoagulation and bleeding risk. Several validated RAMs have been developed to guide prophylactic strategies in cancer-associated thrombosis. Among these, the Khorana Score remains the most widely used, while other models such as Vienna CATS and PROTECHT offer alternative approaches that may complement or refine risk prediction in specific settings^[7]^.

### The Khorana score

The Khorana Score is a prospective, validated RAM originally designed to predict chemotherapy-associated VTE in ambulatory cancer patients. It assigns points based on a combination of tumor type, hematologic parameters, and clinical characteristics:

- Tumor Site: Very high-risk cancers (stomach, pancreas) receive 2 points; high-risk sites (lung, lymphoma, gynecologic, bladder, testicular) receive 1 point.
- Platelet Count: ≥350 × 10^9^/L (1 point)
- Hemoglobin Level or Use of Erythropoiesis-Stimulating Agents: Hemoglobin <100 g/L or ESA use (1 point)
- Leukocyte Count: >11 × 10^9^/L (1 point)
- Body Mass Index (BMI): ≥35 kg/m^2^ (1 point)

A total score of ≥2 is generally considered high risk, identifying patients who may benefit from primary pharmacologic prophylaxis^[6]^.

### Predictive performance

While the Khorana Score has been validated across diverse cancer populations, its performance in ovarian cancer is moderate. Ovarian malignancies are classified as “high risk” and automatically receive 1 point, but many patients may not meet other laboratory or BMI criteria, resulting in an intermediate risk score. Retrospective analyses show that the Khorana Score identifies only a subset of ovarian cancer patients who develop VTE, with sensitivity ranging from 40 to 60% and a positive predictive value of 6–10%. This reflects the unique thrombotic biology of ovarian tumors, which is driven not only by systemic factors but also by local pelvic disease burden, ascites, and surgical interventions that the Khorana model does not capture^[40]^.

### Limitations in ovarian cancer

The Khorana Score’s modest predictive power in ovarian cancer is attributed to several limitations:

- Lack of parameters for tumor bulk, ascites, or cytoreductive surgery, all of which strongly influence thrombosis risk.
- Underestimation of VTE risk in patients receiving anti-angiogenic agents such as bevacizumab or targeted therapies like PARP inhibitors.

Limited adaptability to perioperative and hospitalized settings where different risk dynamics prevail.

Despite these shortcomings, the Khorana Score remains a valuable baseline tool when combined with clinical judgment and disease-specific risk factors^[41]^.

### Vienna CATS model

The Vienna CATS model builds upon the Khorana framework by incorporating biomarkers of coagulation activation, specifically D-dimer and soluble P-selectin levels, in addition to tumor type and blood counts. Elevated D-dimer (>1.44 µg/mL) and soluble P-selectin (>53.1 ng/mL) each confer 1 point, improving predictive accuracy compared to the Khorana Score.

- In validation cohorts, the Vienna CATS model demonstrated C-statistics of 0.73–0.78, outperforming Khorana’s C-statistics of 0.60–0.65.
- In ovarian cancer, high D-dimer levels at diagnosis have been consistently associated with VTE, making this biomarker particularly relevant.

However, the model’s clinical utility is limited by the need for specialized assays (e.g., soluble P-selectin) that are not routinely available in all settings, reducing its feasibility in standard gynecologic oncology practice^[42]^.

### PROTECHT score

The PROTECHT Score was developed to refine VTE risk prediction in ambulatory cancer patients receiving chemotherapy. It adds chemotherapy regimen-related factors, assigning points for the use of platinum-based compounds or gemcitabine, both of which are mainstays in ovarian cancer treatment^[43]^.

- Patients on platinum-based chemotherapy with a baseline Khorana Score of 1 may be reclassified as high risk when using PROTECHT.
- The model has demonstrated improved sensitivity (up to 70%) in certain cohorts of gynecologic cancer patients compared to Khorana alone.

While promising, PROTECHT has not been extensively validated in large ovarian cancer-specific populations, and its performance varies depending on chemotherapy protocols and supportive treatments^[44]^.

### Ovarian cancer-specific considerations: bevacizumab, PARP inhibitors, and thromboprophylaxis in cytoreductive surgery

Ovarian cancer carries one of the highest risks of cancer-associated VTE, a consequence of both tumor biology and treatment-related factors. Beyond the intrinsic procoagulant activity of high-grade serous tumors and the frequent presence of ascites, modern therapeutic strategies – including anti-angiogenic agents, PARP inhibitors, and extensive cytoreductive surgery – further amplify thrombotic risk. These interventions necessitate vigilant risk assessment and tailored thromboprophylaxis protocols to mitigate morbidity and mortality^[45]^.

### Bevacizumab and anti-angiogenic therapy

Bevacizumab, a monoclonal antibody targeting vascular endothelial growth factor, is a cornerstone of first-line and maintenance therapy in advanced ovarian cancer. By inhibiting angiogenesis, bevacizumab disrupts endothelial cell repair and promotes a prothrombotic vascular environment, predisposing patients to both arterial and venous thromboembolic events. Clinical trials such as GOG-0218 and ICON7 reported VTE rates of approximately 7–12% in patients receiving bevacizumab, higher than in those treated with chemotherapy alone. The risk appears greatest during the first 6 months of therapy and is accentuated in individuals with pre-existing cardiovascular comorbidities, bulky disease, or postoperative complications. Although routine primary prophylaxis is not universally recommended during bevacizumab therapy, careful screening for thrombotic risk factors – such as elevated D-dimer levels or a history of prior VTE – can guide the selective use of LMWH or DOACs in high-risk patients^[46]^.

### PARP inhibitors and prothrombotic effects

Poly (ADP-ribose) polymerase (PARP) inhibitors – including olaparib, niraparib, and rucaparib – have transformed the maintenance landscape for BRCA-mutated and homologous recombination-deficient ovarian cancers. While generally well tolerated, emerging pharmacovigilance data suggest a modest but notable increase in VTE risk, particularly with niraparib, which is associated with hematologic toxicities such as thrombocytopenia and anemia. The underlying mechanisms remain unclear but may involve endothelial activation and platelet dysfunction induced by DNA damage response modulation.

Given the extended duration of PARP inhibitor therapy, clinicians should maintain a high index of suspicion for subclinical VTE, especially in patients with baseline risk factors such as obesity, prior thrombosis, or prolonged immobility. Periodic assessment of coagulation markers and prompt evaluation of new-onset symptoms – such as dyspnea, unilateral leg swelling, or chest pain – are essential for early detection^[47]^.

### Thromboprophylaxis in extensive cytoreductive surgery

Primary debulking surgery or interval cytoreduction remains a key therapeutic strategy for advanced ovarian cancer and represents a period of exceptionally high thrombotic risk. The combination of prolonged operative time, large peritoneal surfaces, massive fluid shifts, and postoperative immobility fosters a hypercoagulable state. Reported rates of postoperative VTE in this population range from 10 to 25%, even with standard in-hospital prophylaxis. Current guidelines from the ASCO, American College of Chest Physicians (ACCP), and European Society of Gynaecological Oncology (ESGO) strongly recommend:

- Pharmacologic prophylaxis with LMWH or a DOAC initiated preoperatively or within 12 hours after surgery, provided hemostasis is secure.
- Extended prophylaxis for 28 days postoperatively, as the majority of VTE events occur after hospital discharge.
- Mechanical prophylaxis (e.g., intermittent pneumatic compression) as an adjunct during hospitalization, particularly in patients with temporary contraindications to anticoagulation.

High-risk features – including residual bulky disease, massive ascites, age >60 years, and postoperative complications – warrant strict adherence to extended prophylaxis and careful monitoring for bleeding complications^[48]^.

### Integrating risk across modalities

The convergence of bevacizumab use, prolonged PARP inhibitor maintenance, and aggressive cytoreductive surgery underscores the need for a multimodal thromboprophylaxis strategy in ovarian cancer care. Individualized assessment that incorporates treatment-related exposures, traditional risk models (e.g., Khorana, PROTECHT), and dynamic laboratory markers such as D-dimer or thrombin generation assays can refine decision making. Close collaboration between gynecologic oncologists, hematologists, and supportive care teams is critical to balance the competing risks of thrombosis and bleeding, ensuring safe delivery of these life-prolonging therapies^[48]^.

### Diagnostic strategies and thromboprophylaxis in low-resource environments

Managing VTE in ovarian cancer presents unique challenges in low-resource settings, where advanced imaging, laboratory testing, and consistent access to anticoagulants may be limited. Despite these constraints, early identification and prophylaxis are essential, as delayed diagnosis significantly increases morbidity and mortality. A pragmatic, resource-adapted approach is therefore critical to optimize patient outcomes globally^[45]^.

### Diagnostic strategies

In settings with limited access to CTPA or venous Doppler ultrasonography, diagnosis must rely on a combination of clinical risk assessment, basic laboratory testing, and judicious use of available imaging:

- Clinical Risk Assessment: Structured evaluation using validated tools such as the Khorana Score can help identify high-risk patients even when sophisticated biomarkers are unavailable. Emphasis should be placed on patient history (previous VTE, immobility, and comorbidities), tumor burden, and treatment exposures (e.g., chemotherapy, bevacizumab, and surgery).
- Basic Laboratory Markers: Where possible, D-dimer testing can serve as an adjunctive screening tool. Elevated levels warrant heightened vigilance and prioritization for imaging when available.
- Targeted Imaging: Ultrasound evaluation of symptomatic limbs remains the most accessible diagnostic modality for DVT. For suspected PE, V/Q scans may be substituted for CTPA where feasible^[46]^.

Clinical algorithms should be adapted to prioritize high-yield investigations for patients with moderate-to-high risk, reserving scarce imaging resources for those most likely to benefit.

### Thromboprophylaxis

Thromboprophylaxis strategies must balance efficacy, safety, and feasibility:

- Pharmacologic Prophylaxis: Where LMWH or DOACs are available, standard prophylactic regimens should be employed according to risk stratification. LMWH is often preferred due to its predictable pharmacokinetics and lower need for monitoring, though cost and availability may limit its use. In settings where DOACs are accessible, apixaban 2.5 mg twice daily or rivaroxaban 10 mg once daily may be considered for high-risk ambulatory patients, provided renal function is adequate^[47]^.
- Mechanical Prophylaxis: Intermittent pneumatic compression devices or graduated compression stockings are valuable alternatives or adjuncts where pharmacologic agents are unavailable or contraindicated. Even simple measures such as encouraging early ambulation and leg exercises can reduce thrombotic risk.
- Extended Prophylaxis: For patients undergoing major cytoreductive surgery, extended prophylaxis (up to 28 days) remains desirable; if LMWH is not feasible post-discharge, strategies such as oral DOACs or intensified mechanical measures should be considered^[47]^.

### Implementation considerations

Successful VTE prevention in low-resource environments requires task-shifting and education:

- Healthcare Worker Training: Nurses and community health workers can be trained to recognize early signs of DVT and PE, monitor for anticoagulation complications, and reinforce adherence to prophylaxis.
- Patient Education: Empowering patients to recognize symptoms (leg swelling, pain, dyspnea) and seek timely care is critical. Educational materials should be culturally and linguistically appropriate.
- Resource Prioritization: Hospitals should establish protocols to allocate limited anticoagulants to patients at highest risk, guided by RAMs, surgical complexity, and comorbidities^[48]^.

### Predictive biomarkers in ovarian cancer-associated VTE

The identification of reliable predictive biomarkers for VTE in ovarian cancer has the potential to enhance risk stratification, guide prophylaxis, and improve clinical outcomes. Several biomarkers – most notably D-dimer, TF, and circulating microparticles (MPs) – have been investigated for their utility in predicting thrombotic events, although each comes with limitations in sensitivity, specificity, and clinical applicability^[45]^.

#### D-dimer

D-dimer, a fibrin degradation product, is widely used as a marker of coagulation activation and thrombus formation. Elevated levels are associated with increased VTE risk in cancer patients, including those with ovarian malignancies.

- Predictive Performance: Studies report sensitivity ranging from 80 to 95% for detecting VTE, making it a reliable screening tool. However, specificity is low (30–50%) due to elevation in multiple conditions including infection, inflammation, and post-surgical states.
- Clinical Implications: High D-dimer values can identify patients who may benefit from intensified prophylaxis or early imaging, particularly in high-risk populations undergoing surgery or systemic therapy. Serial measurements may also help monitor dynamic changes in thrombotic risk.
- Limitations: Its low specificity limits use as a standalone diagnostic or predictive test. D-dimer thresholds vary across studies, and assay standardization remains an issue^[46]^.

#### Tissue factor

TF is a transmembrane glycoprotein that initiates the extrinsic coagulation cascade and is often overexpressed in ovarian cancer cells. Elevated TF levels are implicated in tumor-associated hypercoagulability.

- Predictive Performance: Elevated circulating TF has been associated with a two- to three-fold increased risk of VTE in cancer cohorts. Its presence correlates with advanced disease stage, aggressive tumor biology, and poor prognosis.
- Clinical Implications: TF measurement could theoretically identify patients at heightened thrombotic risk who might benefit from prophylactic anticoagulation, particularly during chemotherapy or anti-angiogenic therapy.
- Limitations: TF assays are not standardized for routine clinical use, and circulating levels can be influenced by tissue injury or systemic inflammation, reducing specificity. Limited large-scale validation studies exist in ovarian cancer^[47]^.

#### Circulating microparticles (MPs)

Microparticles are small membrane-bound vesicles shed from activated platelets, leukocytes, or tumor cells. Procoagulant MPs, particularly those expressing TF, contribute to thrombin generation and VTE pathogenesis in malignancy.

- Predictive Performance: Elevated TF-positive MPs have been linked with VTE incidence, with reported HRs ranging from 2.0 to 4.0 in mixed cancer populations. MPs may provide incremental predictive value when combined with clinical risk scores.
- Clinical Implications: MPs could serve as early biomarkers for thrombosis risk, enabling proactive management. They may also offer insight into disease biology and response to therapy.
- Limitations: Measurement of MPs requires specialized flow cytometry techniques, which are not widely available. Pre-analytical variability (sample handling, storage) and lack of standardized thresholds limit reproducibility and clinical translation^[48]^.

#### Integration and current evidence gaps

While D-dimer, TF, and MPs show promise as predictive biomarkers, none currently meet the criteria for routine clinical application in ovarian cancer-associated VTE. Their utility is maximized when combined with validated RAMs, such as the Khorana or PROTECHT scores, allowing a multimodal risk stratification approach. Future research should focus on:

- Standardizing assays and thresholds for TF and MPs.
- Prospective validation in large, ovarian cancer-specific cohorts.
- Integration with clinical and treatment-related variables, including chemotherapy, PARP inhibitors, and anti-angiogenic therapy.

### Future directions

Even with considerable progress in comprehending and handling VTE in ovarian cancer, there are still notable deficiencies in risk assessment, biomarker identification, and the customization of preventive and treatment approaches. Future studies should focus on creating ovarian cancer-specific risk models that integrate genetic, molecular, and clinical factors to improve predictive accuracy beyond general instruments such as the Khorana Score^[6,7]^. A hopeful approach is the incorporation of risk prediction based on biomarkers. Current research on tumor-derived procoagulant elements like TF, cancer procoagulant, and circulating microparticles may produce new biomarkers that can detect high-risk individuals sooner. Likewise, research on inflammatory cytokines, variations in coagulation genes, and tumor genomics may enhance our comprehension of cancer-related thrombosis and aid in precision medicine strategies^[40]^. Progress in anticoagulation treatment also offers new possibilities. DOACs are being used more frequently but necessitate stronger data in gynecologic oncology groups, especially concerning bleeding risk, drug interactions, and effectiveness in particular clinical situations like bowel obstruction or significant ascites. Clinical trials targeting ovarian cancer patients are crucial for directing the safe and effective use of DOACs and for comparing results with traditional treatments such as LMWH^[41]^.

The involvement of immunotherapy and targeted therapies adds further complexity. Medications like PARP inhibitors and anti-angiogenic drugs have transformed treatment options for ovarian cancer but could also influence coagulation pathways. Grasping the prothrombotic or protective influences of these new agents will be crucial for creating thorough thromboprophylaxis strategies suited to modern treatment plans^[42,43]^. Additionally, real-world data and future registries should be utilized to evaluate long-term results of VTE in ovarian cancer patients in various clinical environments. This data can aid in updating guidelines, improve clinical decision making, and highlight disparities in access to thromboprophylaxis or anticoagulation treatment in resource-constrained environments^[44,45]^. Ultimately, models focused on patient-centered care should be emphasized. Integrating patient preferences, enhancing education on VTE signs and prevention, and including thrombosis risk conversations in standard oncology care can empower patients and boost adherence. Digital health tools, such as symptom-tracking applications and telemedicine, can improve the monitoring and early identification of VTE in outpatient groups^[46,48]^.

## Conclusion

VTE is a major and frequently overlooked issue in the treatment of ovarian cancer. Influenced by a complicated interaction of tumor biology, treatment aspects, and individual patient weaknesses, VTE significantly impacts morbidity, delays in cancer treatment, and heightened mortality rates. A thorough comprehension of the pathophysiological processes – like hypercoagulability, endothelial impairment, and inflammatory cascades – is crucial for predicting risk and executing timely interventions. Even with advancements in diagnostic imaging and access to anticoagulant treatments, numerous challenges continue to exist in clinical practice. Timely identification, suitable prevention measures, and tailored management approaches are essential for enhancing results. LMWH and DOAC show effectiveness, but therapy needs careful customization to consider bleeding risk, drug interactions, and patient choices. The perioperative and chemotherapy stages demand careful attention because of increased thrombotic risk.

## Data Availability

Not applicable as this is a perspective.

## Research registration unique identifying number (UIN)

Not applicable as this is a perspective.
